# Surgical Complications of Typhoid Fever, as Observed at U. S. General Hospital, Fort Myer, VA1Read before the Section on Surgery, Buffalo Academy of Medicine, February 5, 1901.

**Published:** 1901-04

**Authors:** Vertner Kenerson

**Affiliations:** Buffalo, N. Y., Attending surgeon, Riverside Hospital; instructor in bacteriology and clinical surgery, University of Buffalo; captain and assistant surgeon, 74th Regiment, N. G., N. Y.; late acting assistant surgeon, U. S. Army


### SURGICAL COMPLICATIONS OF TYPHOID FEVER, AS OBSERVED AT U. S. GENERAL HOSPITAL, FORT MYER, VA.[1]

By VERTNER KENERSON, A. M., M. D., Buffalo, N. Y.,

Attending surgeon, Riverside Hospital; instructor in bacteriology and clinical surgery, University of Buffalo; captain and assistant surgeon, 74th Regiment, N. G., N. Y.; late acting assistant surgeon, U. S. Army.

IN THE spring of 1898, at the outbreak of hostilities between the United States and Spain, I obtained a place as contract surgeon in the army. The contract surgeons were being sent in large numbers south and directly to Cuba at the time I left Buffalo, but in the

1. Read before the Section on Surgery, Buffalo Academy of Medicine, February 5, 1901.

fortunes of war I received a general hospital assignment at Fort Myer, Va. This was a general hospital, situated near Washington, organised to receive typhoid fever patients from the volunteer camps of instruction, and specially from Camp Alger.

At Fort Myer General Hospital, I experienced many interesting phases of typhoid, although not being able to assist in actual field service, and from my notes and charts prepared there, I now gather some points that may be interesting to you, or at least to some among the number here present.

It may be well to outline, in brief, the method of treatment followed with the cases where these complications were noted. Perhaps some of these complications arose on account of the treatment. The patients were taken sick while living in tents, and during their volunteer army service, at Camp Alger, Va. As soon as the true nature of the disease was ascertained, the patients were transferred to Fort Myer, and by as humane a method as possible. In some instances, they were transported in an army ambulance for sixteen miles, over a rough country road. Others were transferred by ambulance for about a mile and a half, and then by trolley car the remainder of the distance. Usually they were brought in such small numbers that they were kept in a recumbent position, but not always.

They were received at all stages of the disease, say from what would seem to be about the fifth day to the thirty-sixth. In no case, except those that were taken sick at the Post (five in number) did the cases come under observation until the fifth day. Some were received with transfer slips, stating they were in their thirty-fifth day of the disease, still in bed with low fever and delirium. The patients, for the most part, were men unacclimated and unused to the surroundings under which they were placed. The conditions were epidemic at Camp Alger.

Their surroundings at Fort Myer were of the best. They were in comparatively small wards, each containing from sixteen to twenty-four beds. One large ward, usually assigned to convalescent patients, contained upward of 300 beds in one room and under one roof. The conveniences were good, the appliances were ample, the nursing was good, and the medical officers were supplied in such numbers that usually one had to care for about sixty cases. Of this number, under ordinary circumstances, not more than one-third would be seriously ill at one time. This arrangement gave each man about twenty-five cases demanding his careful attention; the remainder were convalescents, or were suffering with a mild form of the disease. In general, it was arranged so that an increased force of trained nurses and hospital corps men would serve in the ward where the cases were most serious, and a smaller number in what was known as the convalescent wards. In case a patient had passed the acute stage and been transferred to a convalescent ward, then suffered relapse, he was at once re-transferred to the acute ward.

A majority of the surgeons arrived at Fort Myer prejudiced in favor of the Brand method of treating typhoid fever. They had, however, little or no practical experience in treating such patients as were received there—namely, those who had received no treatment for the first five days and had had a rough journey in arriving at the place of observation. In fact, I do not suppose men experienced in such conditions could have been found. Such conditions had not been present before in our generation. In private practice, with as good nursing and surroundings as were obtainable here, only 2 per cent. of the cases should die, provided the Brand method of treatment could be carried out. There, about 13 per cent. of the cases did die, and this notwithstanding the conditions were made very favorable to the carrying out of the Brand method.

A small minority of the surgeons carried out a purely expectant, symptomatic, course of treatment. Dr. Woodbridge was there, and carried out, in his cases, his own peculiar method of treatment. Under these favorable conditions, but with the class of cases treated, there were no comparisons to be drawn in favor of the Brand method, the expectant method, or the Woodbridge method of treatment. Complications arose among the patients under each method of treatment, and the percentage of deaths and sequelæ was about the same in each case.

It is not my intention in this paper to explain the methods of treatment in detail, to compare the same, or to take up and attempt to settle any disputed points. There will be included in this report, however, certain surgical cases which were operated upon, which were encountered in a group not proven to be typhoid fever, but which occurred with typhoid, or were mistaken for typhoid and sent in as such. There were about 2,300 cases treated. Of these, over 1,000 were typhoid, and about 700 malaria. Of the 1,000 cases, all especially interesting cases were seen by all members of the medical staff, as all met at headquarters every morning before nine o'clock, and all such cases were noted. Sometimes the commanding officer ordered and headed a board of consultation, and always the surgeon in charge of the particular interesting case was very glad to receive professional visits and suggestions, because in many cases under every surgeon's care he saw how hopeless were his own resources. Cases were assigned usually in rotation, except that officers sick with typhoid were sent to a separate ward, where special accommodations had been prepared for them. They, however, could not be given separate rooms, but were placed in small wards, each containing two or three beds.

As patients were discharged, new cases were assigned in rotation, and so each surgeon received approximately his quota of severe typhoid and mild cases, each, of course, with an occasional malarial case, an occasional rheumatic case, and others. Those cases that were evidently surgical were placed in my wards, or those that became surgical were so transferred. Besides these, I cared for and carefully studied sixty-four cases of typhoid. That study and those cases are the basis of the statements contained in this paper. I made personally and observed a positive Widal reaction in each case. This diagnosis was confirmed by an independent observer, Dr. J. J. Curry, of Boston. All cases that did not give the Widal reaction, although they resembled ever so strongly typhoid fever, have been omitted. We were ordered to report the cases where repeated trials failed to give a positive Widal reaction, as remittent malaria, and they are excluded here.

The cases came from regiments hailing from Massachusetts, Rhode Island, New York, New Jersey, Pennsylvania, Ohio, Illinois, Indiana, Missouri, Virginia and West Virginia. The patients were all white and were all volunteers. These 64 cases were under my care, varying from one day to 56 days, averaging 29½ days. Of these cases 8 died; 2 of perforation, 3 of hemorrhage, and 3 of the general severity of the disease, no hemorrhage or perforation being suspected. This is a percentage of 12½ per cent.

No disease is accompanied by so great a number and variety of complications as typhoid fever. Some of the complications named by Flint are: peritonitis, with and following, perforation of the bowel; pleuritis occurring in long and severe cases; pericarditis, endocarditis, meningitis, cerebral hemorrhage—which is very rare—otitis media or externa, cystitis, epididymitis, orchitis, embolism of the arteries of the lower extremities—with gangrene or without—embolism of the pulmonary artery, thrombosis of the femoral vein, with white swelling, thrombosis of other veins, thrombosis of the heart, arteritis, necrosis of bone, periostitis, bedsores, septicemia from bedsores, and other sources, pyemia, with abscess of the lungs, spleen and other parts, erysipelas, furuncles, retropharyngeal abscess, dysentery—chronic, following typhoid,—abscess of the liver, sometimes amebic, the typhoid fever being complicated with amebic dysentery, suppurative arthritis, pyemic or rheumatoid polyarthritis. Among my cases, I had good examples, and will give some details, of the cases having the following surgical complications of typhoid fever: localised and slowly extending general peritonitis, following perforation, in a convalescent patient; parotitis, liver abscess, following complication of amebic dysentery, otitis media, cystitis, orchitis, which went on to suppuration, necessitating unilateral castration, epididymitis; thrombosis of the femoral vein, with white swelling, thrombosis of other veins of lower extremities, bedsores—these were found only on those cases which had been treated for several weeks before being transferred to our care; no bedsores developed in my wards,—erysipelas, furuncles, idiopathic, and furuncles caused by the boring into the skin of a small parasite found on the ground in the Southern States, and known as a "jigger"; rheumatoid polyarthritis, suppurative arthritis.

Perforation of the intestine is liable to occur in one or more ulcers. Perforation occurs in about 1 per cent. of all typhoid cases, according to one authority, and in about 8 per cent. of the deaths from this disease. It is most frequent between the third and fifth weeks of the disease, is rarely caused by the primary sloughing, but is due to secondary ulceration after the separation of the slough. Perforation may be due to rupture of the thin floor of the wall by distension of the intestine with gas, by the impaction of feces, or according to Flint, from the presence of round worms. The opening in the peritoneal coat may be of considerable size, or may be no larger than a pin's point. With intestinal perforation are associated appearances denoting active or diffuse peritonitis.

Peritonitis is accompanied by escape of the gases and other contents of the intestine into the peritoneal sac. In rare instances, peritonitis is circumscribed around the seat of perforation by means of adhesions. Death may occur before sufficient time has elapsed for the appearance of inflammatory exudation. Perforation of the intestine is surgical because in a small percentage of cases of perforation the intestines are inactive and sluggish, and the peristalsis is limited. In these cases, there may be a strictly localised peritonitis, and, if the vitality of the patient is sufficient, operative interference may be advisable. Dr. Frank Hartley, in New York City, has operated upon several such cases—just how many I do not know, and with what percentage of recoveries I do not know. Among my own cases, I had two who had perforation, and both died. One case bid fair to remain localised sufficiently long to operate upon, and I personally believe if operation had been permissible, without moving him from his bed, it could have been done with a chance, small though it may have been, of success. The case was a convalescent, in good condition, recently transferred for purposes of furlough. He had been on full diet (solid food) for a week or ten days, was somewhat constipated, and appeared to be doing well. Suddenly, in the middle of the day, he was taken upon rising beside his chair, with a sharp pain in his left side, midway between the anterior superior spine and umbilicus. The pain was excruciating, he at once went to bed and, on being summoned, I found an acute localised sensitive distension, with rigidity. An hour's delay was necessitated and he was transferred to the operating room where were all appliances. Preparations were pushed, but, at the end of an hour, he was in poor condition; pulse was feeble, high, and breathing was distressed. The peritonitis was no longer localised, but had become general, hence he was returned to his cot, and died that night. An autopsy made by Dr. Zauner, of Philadelphia, showed a general peritonitis, with fecal material and gas, in considerable quantity in the peritoneal sac and a hole in the intestine about as large as the lead in an ordinary pencil.

The next complication I wish to note is that of parotitis, which is rare and involves one or both of the parotid glands. It leads to notable enlargement, and the appearance is like that of ordinary parotitis, or mumps; but, unlike the affection just named, in the great majority of cases suppuration takes place, and not infrequently there is more or less sloughing of the areolar tissue. This complication adds to the danger and retards convalescence, but is not to be regarded as a critical event, and may occur at any time of the febrile period, or during convalescence. Discharge of pus sometimes occurs into the meatus auditorius. This complication occurs in typhus, according to Flint, as well as typhoid. The cases of parotitis were interesting, and there were four in the list, only one double, the others involving the right side of the face.

The case of Private Denton, whose chart and outline of history is among those presented, was typical. He had been under my care for twelve days, with a temperature varying from 101° to 104°—with one drop to normal which led to a suspicion of hemorrhage, but none materialised—when on the 12th day, the right side of his face began to swell and became very tender. His father was present after the third day of his sickness and he said the boy had had mumps on both sides when a child. I simply state this fact as one of the surgeons present, Dr. Bradfield, a man of wide experience, and who had served in a surgeon's capacity in 1861-5, stated that the complication of parotitis did not occur in adults who had had mumps in infancy.

Denton's treatment, from time of admission to time of development of the parotitis, had been the Brand treatment unmodified. He had received from one to two plunge baths a day for any temperature of 103° or higher. After the development of parotitis, as above stated on the 12th day, his treatment was modified and he was sponged instead of being plunged. As soon as the parotitis developed, his face was poulticed with cloths wrung out of 1-80 carbolic solution, changed often enough to be kept warm. These were continued until fluctuation occurred, and then the gland was laid open freely under an anesthetic. As will be seen by looking at this chart, the complication raised his temperature for two or three days, until it was opened. On the fourth day of the progress of the parotitis, his temperature fell $6\frac{1}{4}$°, and again the stools were watched carefully for hemorrhage, but none was found. The face, however, had become pretty well blanched by the application of the carbolic solution. Possibly the solution may have been used stronger than ordered. At any rate, I could not find any other good explanation for the sudden and great fall of temperature, unless it was from the coal tar poisoning from the long continued or too strong use of carbolic acid. The solution was changed, and his chart promptly resumed a low grade of fever, which continued for seven days more and then fell to normal. Parotitis is stated by Osler and Pepper to be a serious and dangerous complication and as occurring in only very sick cases. None of the cases observed by me died.

The occasional occurrence of thrombosis of the iliac or femoral vein on one or both sides is not rare, and is attended with swelling and pain of the corresponding limb. This complication rarely leads to evil consequence, but sudden death has been known to occur, caused by an embolus detached from the thrombus occluding the pulmonary artery.

In all cases, with such profound prostration as was present in most of these sixty-four cases, the patients remained so still in bed that they did not in many cases make any voluntary movement with the legs for some days or weeks. Following such inaction and low vitality, they, for the most part, had some difficulty when first allowed to swing their feet out of bed, with the lower extremities. Almost without exception, they complained during the first week of convalescence of pain, and swelling was frequently observed. This, however, was not due to thrombosis of the femoral, but to muscular inactivity and, if I may use the term, temporary atrophy. I saw, however, one case of apparently complete occlusion of the femoral vein, following a prolonged and severe case of typhoid fever. This patient, Chenoworth, a private of the 3d Missouri Regiment, had a typical occlusion of both femorals. The veins were discernible and easily palpable, whipcord like, and were so when he was discharged and sent home. The lymphatics were also evidently occluded, although no such demonstration was possible as in the case of the veins. So long as the patient remained in bed, his legs would be of proper size, but, when he was allowed to sit up, and latter when he was allowed to stand and walk, his legs promptly became swollen and at first very painful. Collateral circulation was very slowly established and complete recovery followed. The legs were bandaged, gentle massage was given below the knee, and no manipulation was allowed near the femoral opening for fear of dislodging the upper end of the clot and causing disastrous results. Chenoworth's case was the only one, in my series, where the veins occluded could be felt on both sides, and who completely recovered.

Private Connell, who when admitted was serving in the hospital corps, having been detailed to that duty from his regiment, which was originally the 5th Massachusetts, presented an interesting complication. The patient was past the average age of enlisted men, and was probably 38 or 40 years old. He was a native of Ireland, but had been a resident of Massachusetts for fifteen years. He was admitted to the wards of Dr. Pond with the transfer slip reading typhoid fever, and he gave the Widal reaction. He had been under treatment about seven days, his temperature running an irregular, but not septic chart, but as typical as most of our charts were of the text-book pictures of typhoid charts, when Dr. Pond discovered a fluctuating yet very resistant tumor below the ensiform cartilage. A consultation was held, and, without arriving at a diagnosis, the case was ordered transferred to my ward, and an exploratory diagnostic operation was determined upon. Ether was given, and I aspirated over the tumefaction and there obtained a quantity of pus. This, in one way, cleared the diagnosis, so I made a liberal incision into the most prominent part of the tumor and explored carefully with my finger. The abscess cavity was in the right lobe of the liver and practically involved the whole lobe. The adhesions bound the whole lobe of the liver very firmly to the body wall, hence I was careful not to break up the adhesions and enter the abdominal cavity. The abscess was filled with a soft, slimy, thick, viscid pus, not particularly foul smelling, but tenacious and not fully disintegrated. The abscess cavity was not walled, but uneven, and it would appear as if the liver had begun to decay in the center and had slowly eaten toward the capsule, decomposing faster in the liver tissue, and slower where there was a retaining fibrous tissue. It could be scooped out almost in cupfuls. The large extent of the abscess and the very poor condition of the patient forbade anything but necessary manipulation. The cavity was washed out with sterile salt solution and the wound and cavity packed with a Mikulicz bag of iodoform gauze filled, after placing, with crumpled sterile gauze; the patient was stimulated, and put to bed with plenty of heat.

Specimens of pus were examined by Dr. J. J. Curry, who found the material free from bacteria, but when examined on a warm stage, under light pressure and with a low power objective, the ameba of amebic dystentery were found in large numbers. These were kept alive for a day or two. Meanwhile, careful examinations of the stools were made and the ameba were found in plenty. The man's condition was precarious at the time of operation and he lived about forty-eight hours afterward. His case was the only one of amebic dystentery and complicating amebic abcesss of the liver reported. He had served with his regiment, with the same army corps, had had the same food, drank the same water, and had been taken sick while serving in the division hospital corps, and while caring for incipient typhoid cases, yet no other cases were reported.

The ameba coli was first accurately described by Losch in 1873. It was a round or irregularly shaped cell, about .02 or .04 mm. in diameter, consisting of a clear outer zone of protoplasm and of granular substance within. In the quiescent state, the ameba is round, but ordinarily it is in active movement, projecting pseudopods on every side, often several at a time, but usually only one or two. When surrounding conditions are not favorable to its life, the organism becomes encysted. In this condition, it is round or oval and quiescent, and is surrounded by a resistant outer covering.

The especially interesting thing about Connell's case was that his disease was tropical dysentery mistaken for complicating typhoid fever, occurring in a man from a northern state, then present in Virginia, having been there less than a month, aad where the temperature was 80-90°, with the nights cool. He was taken sick in July,

1898. We examined many other cases when the dysentery was a marked symptom, but did not find the ameba coli again. An ameba, which at any rate is similar to this one, is found occasionally in the swampy land water in this vicinity. In October of 1900, I saw some specimens in the hands of Dr. Williams and Dr. Barrows at the pathological laboratory of the University of Buffalo. I suppose this needs simply a proper climate, a weakened constitution and an entrance into the system to bring about a dysentery, which was for many years a mysterious uncontrollable and usually fatal disease and was known as tropical dysentery.

In referring to intestinal hemorrhage as a surgical complication of typhoid, I must needs explain that these were surgical cases simply because we treated them surgically—namely, with intravenous injection of sterile normal salt solution. When any sudden fall in temperature indicated a possible hemorrhage, all plunge bathing was at once discontinued, sponge baths were substituted and were given for any temperature of 103° or worse, opium was given, and, if no vomiting or undigested curds had been observed in the stools, the milk was scalded and served cold, as before, the frequency of feeding being made three instead of two hours. If the stools following the drop in temperature showed the characteristic tarry mass or blood, pure and simple, or if the unmistakable collapse followed and the pulse became weak, thready and high, the diagnosis of hemorrhage was confirmed.

Some of these cases of hemorrhage showed very few untoward symptoms. They were simply given one-third of a grain of opium, the diet modified for a day or two, sponge baths in place of plunge baths were continued, and they went forward to convalescence. Some other cases, severe in character, discouraging to the general look, were treated with intravenous injections of salt solution. The median basilic vein was opened, a canula was inserted, and sufficient salt solution was injected to bring the pulse up, so that it was a palpable quantity. This required, in every case, one pint, and in two cases, three pints. The veins were not any harder to find, secure and open, than they would be in any exsanguinated person. Of these seven cases, all severe, two lived and five died. Of the five who died, two were members of the Niagara Falls Company, and were desperately sick when admitted to my wards. They were the famous cases of Stahl and Stricker, who died a few hours after admission and were sent home after death. The government does not furnish a new uniform for deceased soldiers who have not had forethought enough to draw one, and these two men came to the general hospital in suits in which they had been digging trenches when they were taken sick, they having drawn only one suit. While they were at Fort Myer, they had been bathed every four hours, put into clean sheets a dozen times, but when they died and we were ordered to send them home, they were dressed in their own suits, which were not clean. When they were received in this condition at Niagara Falls, a great howl was raised, a public indignation meeting was held, inflammatory speeches were made, money was raised, and a committee of public safety was ordered to come and investigate us. I met the committee, which was an extremely sensible one, composed of Major Butler (now mayor of Niagara Falls), and one of the ministers there, who was sensible, and yet who wanted to know why those things were necessary. The cases were both desperate cases and should not be counted in this consideration. The percentage there is improved, counting the five cases—two recovered and three died—and these were when repeated hemorrhages had occurred, when the general condition was poor, pulse weak, and nothing in their favor.

Dr. Woodbridge treated one case, which was not transferred to me, by subcutaneous injections of salt solution, but the case had to be counted in his mortality list, which I think was the same as my own, 12½ per cent. It was thought that the normal salt solution, instilled as I have suggested, abated the intensity of the disease in the two cases mentioned which recovered, and I used it after consultation with other members of the staff and Major Davis in two cases that were desperately ill, with marked nervous symptoms, and yet with weak pulse. I instilled in each case a pint and a half, with the result that the character of the pulse was improved, the nervous symptoms were improved, and a temporary improvement was noted in the charts, but both cases died.

I wish to digress here to head off a repetition of some criticism which I gladly received from Drs. Eugene and Chauncey P. Smith when I read my last paper before this body—namely, that salt solution properly introduced at a right temperature (110° to 120°) in proper cases never kills *per se*. I have made a careful search of the literature by means of the indices and periodicals, and can find no mention made of the matter, and must decline to believe that in cases of collapse from loss of blood, sudden serious impoverishment of the blood as from some loss—probably small—and a high grade of fever that the intravenous injection of salt solution can be of any injury, but will be of direct advantage. The case in which this criticism was evoked was one in which serious loss of blood and lymph was encountered, and was expected, and preparation made accordingly, and when collapse came prompt instillation was made, and the criticism was that the salt solution killed the child, hence should not have been used.

There were several other cases where, for hemorrhage, intravenous instillation of salt solution was practised, but they did not come under my personal care except for the operative procedure, which are not included in this paper. Dr. Baker, of Washington, D. C., was in charge of several of these cases, which all did well immediately, but whether they went on serenely to convalescence or not I do not know. I saw in consultation two cases of otitis media, complicating typhoid. They were both in men who were uncleanly in their habits, and both appeared during the severity of the disease. Both cases had far advanced, and were only detected when rupture of the drum and exudate of pus announced the condition. Both cases showed a staphylococcus pus and no typhoid bacillus. I think these cases were coincident, caused by weakened condition and uncleanly habits.

Cystitis after prolonged confinement in bed with great prostration and more atonic than septic, was seen in three cases. There was no treatment adopted except to hurry the convalescence, push the food and stimulation, and give benzoate of sodium in sufficient doses to get and keep the urine acid, or nearly so. The patients suffering from cystitis were given massage, urged to get up and out into the sun. Bladder washing was not found necessary in any case. Several cases of orchitis were developed in patients where no history of gonorrhea could be obtained, and the history in each case was considered reliable. In one case the condition continued for two weeks and, a small fluctuating spot appearing near the skin of the scrotum, it was opened, considerable pus discharged and continued to discharge. The sinus opened led to many others. Sensations on pressure were no longer referred to the cord, but simple tenderness as over any abscess supervened. It was concluded the entire testicle had become disintegrated, and I accordingly removed the organ by the usual procedure. The spermatic cord was not found involved. The patient made an uneventful recovery, was up and out in a few days, and shortly after discharge from hospital was arrested for rape.

Cases of erysipelas developed, and were isolated, but were not transferred to my wards, and none of them were complicated by pus formations. Numerous cases of extensive involvement of the skin by furuncle were noted, and were due, I suppose, to the general atony following the disease. They appeared on all parts of the body, but were not partial to the hands and face, preferring the limbs and body. Among these cases were two of especial interest, as their etiology involved what was to me a new process. These patients were received in a convalescent stage from one of the Southern camps. They had been quartered in camps for some time and had slept on the ground or upon their blankets when not displaced. They had then been taken sick, their disease had taken its course, and while sick in hospitals had, I believe, been sleeping on hospital cots. When received at Fort Myer they were covered with little boils apparently all over their backs. No space as large as a silver dollar but that had a small or large a-coming or a-gone furuncle. One of our surgeons from Cincinnati, who served as hospital steward in the army in 1864-5, and who was still active, recognised the condition at once, and, squeezing out one of the festers, showed that it was the larvæ of a "jigger," a small round insect that delights to bore into the skin of men sleeping on the ground in the Southern States and deposit its eggs. These in due time become hatched and a foreign body, and in the run-down condition in which a convalescent typhoid found himself easily infected. The treatment was the popping out of these little grub-like animals and syringing out each little cavity with peroxide of hydrogen. The cavities usually healed kindly. When I saw those men's backs, I for the first time appreciated the exclamation I had heard many times before but never knew the meaning of. The exclamation was, "Well, I'll be jiggered." Judging from his suffering and appearance, no one would ever be "jiggered" if he could help himself.

Finally, I cannot close the paper without a word for my associates and superior. They were all gentlemen, men of wide personal experience, both in the general experience and professional attainment. Dr. Baker, of Washington, was a keen observer, careful and painstaking. He was scientific, and had had a considerable acquaintance with the flats around Washington and their malarial tendencies, and was of great assistance in blood examinations.

Dr. Zauner, of Philadelphia, was for many years connected with the board of health in New York City, and was of great assistance, and by his keen observation kept us from epidemics of measles, erysipelas and smallpox.

Dr. J. J. Curry, of Boston, was one of Dr. Councilman's and Dr. Mallory's proteges, and although argumentative, was of inestimable value.

Finally, Major Davis, who is known to many of you here, was the commanding officer. He will be remembered by the University of Buffalo men as a student in '55. While here he was, I think, as he was when I knew him in New York, an unobtrusive keen observer and accurate analysist of new methods and always a hard worker and full of resource. He passed almost thirty years of his life in the service of the United States army. He knew intimately all the requirements of the service, and red tape to him was just simply a measuring rod to lay off how much and when needed, and cut it off. He was a loyal and generous commanding officer, and we all reverenced him, respected him and obeyed him willingly, and always with pleasure and advantage to ourselves.

186 Allen Street.

